# Aberrant DNA Methylation of *OLIG1,* a Novel Prognostic Factor in Non-Small Cell Lung Cancer

**DOI:** 10.1371/journal.pmed.0040108

**Published:** 2007-03-27

**Authors:** Romulo M Brena, Carl Morrison, Sandya Liyanarachchi, David Jarjoura, Ramana V Davuluri, Gregory A Otterson, David Reisman, Selina Glaros, Laura J Rush, Christoph Plass

**Affiliations:** 1 Department of Molecular Genetics, The Ohio State University, Columbus, Ohio, United States of America; 3 Department of Pathology, The Ohio State University, Columbus, Ohio, United States of America; 2 Department of Molecular Virology, Immunology and Medical Genetics, Division of Human Cancer Genetics, The Ohio State University, Columbus, Ohio, United States of America; 4 Division of Biostatistics, College of Medicine and Public Health, The Ohio State University, Columbus, Ohio, United States of America; 5 Department of Internal Medicine, Division of Hematology and Oncology, College of Medicine and Public Health, The Ohio State University, Columbus, Ohio, United States of America; 6 Department of Internal Medicine, Division of Hematology and Oncology, University of Michigan, Ann Arbor, Michigan, United States of America; 7 Department of Veterinary Biosciences and the Comprehensive Cancer Center, The Ohio State University Columbus, Ohio, United States of America; University of Pittsburgh School of Medicine, United States of America

## Abstract

**Background:**

Lung cancer is the leading cause of cancer-related death worldwide. Currently, tumor, node, metastasis (TNM) staging provides the most accurate prognostic parameter for patients with non-small cell lung cancer (NSCLC). However, the overall survival of patients with resectable tumors varies significantly, indicating the need for additional prognostic factors to better predict the outcome of the disease, particularly within a given TNM subset.

**Methods and Findings:**

In this study, we investigated whether adenocarcinomas and squamous cell carcinomas could be differentiated based on their global aberrant DNA methylation patterns. We performed restriction landmark genomic scanning on 40 patient samples and identified 47 DNA methylation targets that together could distinguish the two lung cancer subgroups. The protein expression of one of those targets, *oligodendrocyte transcription factor 1 (OLIG1),* significantly correlated with survival in NSCLC patients, as shown by univariate and multivariate analyses. Furthermore, the hazard ratio for patients negative for OLIG1 protein was significantly higher than the one for those patients expressing the protein, even at low levels.

**Conclusions:**

Multivariate analyses of our data confirmed that OLIG1 protein expression significantly correlates with overall survival in NSCLC patients, with a relative risk of 0.84 (95% confidence interval 0.77–0.91, *p* < 0.001) along with T and N stages, as indicated by a Cox proportional hazard model. Taken together, our results suggests that OLIG1 protein expression could be utilized as a novel prognostic factor, which could aid in deciding which NSCLC patients might benefit from more aggressive therapy. This is potentially of great significance, as the addition of postoperative adjuvant chemotherapy in T2N0 NSCLC patients is still controversial.

## Abstract 2

Christopher Plass and colleagues find that*OLIG1* expression correlates with survival in lung cancer patients and suggest that it could be used in deciding which patients are likely to benefit from more aggressive therapy.

## Editors' Summary

**Background.:**

Lung cancer is the commonest cause of cancer-related death worldwide. Most cases are of a type called non-small cell lung cancer (NSCLC). Like other cancers, treatment of NCSLC depends on the “TNM stage” at which the cancer is detected. Staging takes into account the size and local spread of the tumor (its T classification), whether nearby lymph nodes contain tumor cells (its N classification), and whether tumor cells have spread (metastasized) throughout the body (its M classification). Stage I tumors are confined to the lung and are removed surgically. Stage II tumors have spread to nearby lymph nodes and are treated with a combination of surgery and chemotherapy. Stage III tumors have spread throughout the chest, and stage IV tumors have metastasized around the body; patients with both of these stages are treated with chemotherapy alone. About 70% of patients with stage I or II lung cancer, but only 2% of patients with stage IV lung cancer, survive for five years after diagnosis.

**Why Was This Study Done?:**

TNM staging is the best way to predict the likely outcome (prognosis) for patients with NSCLC, but survival times for patients with stage I and II tumors vary widely. Another prognostic marker—maybe a “molecular signature”—that could distinguish patients who are likely to respond to treatment from those whose cancer will inevitably progress would be very useful. Unlike normal cells, cancer cells divide uncontrollably and can move around the body. These behavioral changes are caused by alterations in the pattern of proteins expressed by the cells. But what causes these alterations? The answer in some cases is “epigenetic changes” or chemical modifications of genes. In cancer cells, methyl groups are aberrantly added to GC-rich gene regions. These so-called “CpG islands” lie near gene promoters (sequences that control the transcription of DNA into mRNA, the template for protein production), and their methylation stops the promoters working and silences the gene. In this study, the researchers have investigated whether aberrant methylation patterns vary between NSCLC subtypes and whether specific aberrant methylations are associated with survival and can, therefore, be used prognostically.

**What Did the Researchers Do and Find?:**

The researchers used “restriction landmark genomic scanning” (RLGS) to catalog global aberrant DNA methylation patterns in human lung tumor samples. In RLGS, DNA is cut into fragments with a restriction enzyme (a protein that cuts at specific DNA sequences), end-labeled, and separated using two-dimensional gel electrophoresis to give a pattern of spots. Because methylation stops some restriction enzymes cutting their target sequence, normal lung tissue and lung tumor samples yield different patterns of spots. The researchers used these patterns to identify 47 DNA methylation targets (many in CpG islands) that together distinguished between adenocarcinomas and squamous cell carcinomas, two major types of NSCLCs. Next, they measured mRNA production from the genes with the greatest difference in methylation between adenocarcinomas and squamous cell carcinomas. *OLIG1* (the gene that encodes a protein involved in nerve cell development) had one of the highest differences in mRNA production between these tumor types. Furthermore, three-quarters of NSCLCs had reduced or no expression of OLIG1 protein and, when the researchers analyzed the association between OLIG1 protein expression and overall survival in patients with NSCLC, reduced OLIG1 protein expression was associated with reduced survival.

**What Do These Findings Mean?:**

These findings indicate that different types of NSCLC can be distinguished by examining their aberrant methylation patterns. This suggests that the establishment of different DNA methylation patterns might be related to the cell type from which the tumors developed. Alternatively, the different aberrant methylation patterns might reflect the different routes that these cells take to becoming tumor cells. This research identifies a potential new prognostic marker for NSCLC by showing that OLIG1 protein expression correlates with overall survival in patients with NSCLC. This correlation needs to be tested in a clinical setting to see if adding OLIG1 expression to the current prognostic parameters can lead to better treatment choices for early-stage lung cancer patients and ultimately improve these patients' overall survival.

**Additional Information.:**

Please access these Web sites via the online version of this summary at http://dx.doi.org/10.1371/journal.pmed.0040108.

- Patient and professional information on lung cancer, including staging (in English and Spanish), is available from the US National Cancer Institute
- The MedlinePlus encyclopedia has pages on non-small cell lung cancer (in English and Spanish)
- Cancerbackup provides patient information on lung cancer
- CancerQuest, provided by Emory University, has information about how cancer develops (in English, Spanish, Chinese and Russian)
- Wikipedia pages on epigenetics (note that Wikipedia is a free online encyclopedia that anyone can edit)
- The Epigenome Network of Excellence gives background information and the latest news about epigenetics (in several European languages)

## Introduction

Lung cancer is the leading cause of cancer-related death worldwide [1]. It is estimated that over 1.2 million people are diagnosed with lung cancer annually, and 1.1 million die from the disease [2]. Despite intensive research over the past decades, the five-year survival of lung cancer patients remains poor [3]. Currently, the most accurate prognostic factor for patients with non-small cell lung cancer (NSCLC) is tumor, node, metastasis (TNM) clinico-pathologic staging [4]. Nevertheless, patients with early-stage lung cancer exhibit a wide spectrum of survival, indicating the need for additional prognostic parameters to better predict the outcome of the disease [5]. Thus, much effort has been dedicated to identifying molecular markers that might improve the classification of NSCLC. Such markers not only should give prognostic information, but could help identify patients that would benefit from novel therapeutic strategies or, alternatively, those for which additional treatment is not needed. A recent example of the utility of such markers is the identification of gene expression profiles that predict high risk of recurrence of localized lung cancer [6].

Over the past decade it has become evident that the cancer genome is marked by epigenetic modifications that contribute to the deregulation of transcription profiles [7,8]. Of particular interest is the observation that certain genes demonstrate differential susceptibility to epigenetic deregulation. That is, some genes are targeted for promoter methylation in only some tumor types [9,10], while others are common targets for DNA methylation in several types of neoplasias [11]. Thus, a genome-wide scan for DNA methylation in NSCLC could uncover new clinically relevant molecular targets.

We analyzed primary human lung tumor samples via restriction landmark genomic scanning (RLGS) [12] to identify DNA sequences differentially methylated between the two major NSCLC subgroups, adenocarcinomas and squamous cell carcinomas (SCCs). We uncovered promoter methylation patterns characteristic for both NSCLC subtypes and describe a novel marker, *oligodendrocyte transcription factor 1 (OLIG1)*, whose expression correlates with overall survival in NSCLC patients, as validated by univariate and multivariate analyses.

## Methods

### Procurement of Primary Human Tissue Samples

Primary lung cancer and adjacent tumor-free tissue samples were procured through the Cooperative Human Tissue Network at The Ohio State University James Cancer Hospital and The University of Michigan following approved Internal Review Board protocols. Consent from participants was waived under CFR 46 subpart A. A total of 70 snap-frozen matched tumor-free/adenocarcinomas and 70 snap-frozen matched tumor-free/SCCs were procured. For immunohistochemical studies, two lung tissue microarrays (TMAs) were generated. All specimens included in these arrays were cored from formalin-fixed paraffin-embedded tissue blocks. TMA1 comprised 67 adenocarcinomas, 82 SCCs, and six tumor-free lung samples arrayed in quadruplicate. TMA2 comprised 74 adenocarcinomas and 79 SCCs arrayed in triplicate. DNA isolated from peripheral blood mononuclear cells procured from random cancer-free donors was utilized as a negative control for DNA methylation. In an effort to facilitate the tracking of which tumor specimens were utilized only once in this study versus those employed in several experimental approaches, each specimen has been assigned a unique identifier. Adenocarcinomas are denoted as Adeno followed by a number, while squamous cell carcinomas are denoted as SCC followed by a number.

### RLGS

RLGS was performed as previously described [13]. To avoid potentially confounding factors, such as age-related DNA methylation [14], samples were selected so that gender, race, and age range would be comparable between the adenocarcinoma and the SCC tumor subsets (Table S1). RLGS profiles of primary tumors and tumor-free lung from the same patient were superimposed and visually inspected for differences in the presence and/or intensity of radiolabeled fragments. The investigator performing the analysis was blinded as to the cancer subtype of each sample. The use of control tissues derived from the same patient as the tumor sample ensured that DNA polymorphisms that might be present at any of the restriction enzymes' recognition sites would not introduce a bias in the analysis.

### Identification of RLGS Fragments

RLGS fragments of interest that had not already been identified in our laboratory were cloned with the aid of either a human NotI–EcoRV or a human AscI–EcoRV plasmid library, as previously described [13,15,16]. Alternatively, a PCR-based approach was employed to identify RLGS fragments not present in the libraries [16].

### RNA Isolation and Quantitative Real-Time PCR

Total RNA from primary human samples and human lung cancer cell lines was isolated and purified as previously described [17]. RNA integrity was assessed with the Agilent 2100 Bioanalyzer using an RNA 6000 LabChip kit (Agilent Technologies, http://www.agilent.com). Only samples that showed high level of RNA integrity were used for reverse transcription [18]. For each sample, 1 μg of total RNA was reverse transcribed using oligo dT (Invitrogen, http://www.invitrogen.com), as previously described [19]. Given the fact that OLIG1 is an intronless gene, regular PCR was performed on DNAseI-treated but not reverse-transcribed RNA samples to ensure that no DNA contamination was present in the RNA extracts. Quantitative *OLIG1* expression was measured using SYBR Green I (Bio-Rad, http://www.bio-rad.com) in an iCycler (Bio-Rad). *Calcium/calmodulin-dependent protein kinase kinase 2 (CAMKK2)* was used as internal control [19]. Primer sequences and PCR conditions for all genes described in this study are listed in Table S2.

### Combined Bisulfite Restriction Analysis and Combined Bisulfite Restriction Analysis Coupled with the Agilent 2100 Bioanalyzer Platform

Combined bisulfite restriction analysis (COBRA) was performed on *BAHD1* and *DMRTA1* as previously described [20]. Briefly, 181-bp and a 218-bp fragments from the *BAHD1* and *DMRTA1* genes, respectively, were amplified by PCR from bisulfite-treated DNAs. The PCR products were purified and digested with 10 U of BstUI (New England Biolabs, http://www.neb.com) at 60 °C for 4 h. The digested samples were electrophoresed in an 8% polyacrylamide gel and visualized via ethidium bromide staining. Primers and PCR conditions are listed in Table S2.

Combined bisulfite restriction analysis coupled with the Agilent 2100 bioanalyzer platform (Bio-COBRA) was performed as previously described [19,21] on 41 out of the 59 samples utilized to assess deletions at the *OLIG1* locus. The reduction in the number of samples analyzed by Bio-COBRA was due to limitations in the amount of tumor DNA available from some specimens. Briefly, genomic DNA was isolated from human primary lung tumors, which was then mechanically sheared and bisulfite treated [22]. Bisulfite-treated DNAs were PCR amplified with *OLIG1*-specific primers (Table S2), purified, and digested with 10 U of BstUI (New England Biolabs) at 60 °C for 4 h. We electrophoresed 5 μl of the digestion reaction in an 8% polyacrylamide gel and visualized the digestion patterns via ethidium bromide staining. We loaded 1 μl of each digestion product onto a DNA 500 LabChip and assayed them using the Agilent 2100 Bioanalyzer. Chromatograms were visually examined, and the raw data generated from the assay was plotted to obtain the fluorescence values for each of the digestion fragments. The methylation percentage for each sample was calculated as follows: fluorescence of methylated products/(fluorescence of methylated products + fluorescence of unmethylated product).

### OLIG1 Luciferase Assay

Using primers tagged with *Not*I or *Eco*RV sequence tails, four *OLIG1* constructs were generated by PCR (Table S2). The constructs were directionally cloned into a pGL3-Basic vector (Promega, http://www.promega.com) modified to contain NotI and EcoRV restriction sequences in its multiple cloning site. A549 cells were plated at a density of 2 × 10^4^ cells/35-mm well in RPMI-1640 medium (Cellgro, http://www.cellgro.com) supplemented with heat-inactivated 10% FBS (Cellgro) the day before transfection. The next day, cells were transfected as previously described [17]. A promoterless pGL3-Basic vector was used as the negative control for expression and a pGL3-Basic vector containing the E2F3a promoter was used as the positive control. Renilla luciferase was used as the transfection efficiency-normalizing factor. Luciferase activity was measured using the Dual Luciferase assay system (Promega). All measurements were performed in triplicate and the experiment was repeated three times.

### 5-aza-2′Deoxy-Cytidine Treatment of Human Lung Cancer Cell Lines

Human NSCLC cell lines A549 and H1299 were cultured for two days and then treated with 1 μM of 5-aza-dC (Sigma-Aldrich, http://www.sigmaaldrich.com) for 48 and 72 h as previously described [17]. After treatment, total RNA was isolated as previously described [17].

### Assessment of *OLIG1* Deletions in Primary Tumors

DNA was isolated from snap-frozen tissues as previously described [13]. The DNAs were sheared and diluted to a final concentration of 20 ng/μl. Real-time PCRs were performed using SYBR Green I (Bio-Rad) in an iCycler (Bio-Rad). *CAMKK2* was used as internal control. All reactions were performed in triplicate. The *OLIG1* threshold crossing (Ct) value for each sample was normalized to that of its internal control by subtracting the *OLIG1* Ct from the *CAMKK2* Ct. The *OLIG1* level in the tumor samples was calculated by the ΔCt method, setting the normalized *OLIG1* values obtained from the matching tumor-free DNA to 1. A sample was considered to harbor a deletion at the *OLIG1* locus if reduction of *OLIG1* at the DNA level was assessed to be >25% compared to its matching normal control [23]. The overall comparison for the frequency of deletions between the adenocarcinomas and the SCCs was assess by a one-tail Z-ratio and considered significant if the result of the test was *p* ≤ 0.050.

### Bisulfite DNA Sequencing

Bisulfite DNA sequencing was performed on two adenocarcinomas, two SCCs, and the four tumor-free lung tissues from the same patients, as previously described [17]. We sequenced eight to ten individual clones per sample. Primer sequences and PCR conditions are listed in Table S2.

### Immunohistochemical Staining and Scoring of Primary Lung Tumor Tissue Arrays and a Lung Cancer Cell Line Array

Immunohistochemical staining of human primary lung tumor samples was performed on TMA1 composed of formalin-fixed, paraffin-embedded specimens. Each specimen was present four times in the array [24]. The array contained 67 different adenocarcinomas, 82 different SCCs, and six tumor-free lung samples. Brain tissue cores were included as positive controls for *OLIG1* staining (Table S3 lists the clinical features of the specimens included in this array). Validation of the immunohistochemistry results generated from TMA1 was performed on an independent sample set (TMA2). This sample set comprised 74 formalin-fixed, paraffin-embedded adenocarcinomas and 79 formalin-fixed, paraffin-embedded SCCs arrayed in triplicate (Table S4 lists the clinical features of the specimens included in this array). A mouse monoclonal anti-OLIG1 antibody (R&D Systems, http://www.rndsystems.com) was used at 1:1,000 dilution for immunohistochemical detection. Antibody binding was detected by incubating the slides with a secondary polyclonal anti-mouse IgG antibody (Amersham Biosciences, http://www.amersham.com). Positive staining was visualized by incubating the slides with diaminobenzadine (Sigma-Aldrich).

The slides were examined by an experienced lung pathologist (CM) and reviewed by the primary investigator (RMB). The evaluation of the immunohistochemical results was performed as follows: each tissue core was assigned an “OLIG1 index score,” calculated on two parameters, percent of positive (stained) cells in the tumor epithelium and intensity of staining [25]. Each parameter was subdivided into three categories: for percent of positive cells, 0%–10% was assigned a value of 1; 10%–50% was assigned a value of 2, and >50% was assigned a value of 3. For the intensity of staining, no staining was assigned a value of 1, weaker than normal lung staining was assigned a value of 2, and staining as strong as normal lung was assigned a value of 3 [26]. The OLIG1 index for each core was then calculated by multiplying the value assigned to each parameter. In order to ensure the accurate assessment of OLIG1 protein expression in each tumor, either three or four cores of the same sample were placed in the tissue arrays. This design helped overcome the problem of tumor heterogeneity, which could affect the results depending on what area of tumor is cored. The final OLIG1 index score for each sample was determined by taking the average of the indexes given to each individual core.

OLIG1 protein levels were also assessed via immunohistochemistry in H1299 cells treated with 1 μM of 5-aza-dC for 48 and 72 h. After treatment, the cells were collected, embedded in agar pellets, and fixed in formalin as previously described [17]. After fixation, each pellet was cored twice and placed on a single slide to create a cell line array. OLIG1 protein detection was performed following the same protocol utilized on the human primary tissue arrays as previously described.

### Statistical Analysis

In order to identify candidate RLGS loci that show frequent methylation in one tumor subtype compared to the other, proportions of methylation in the two groups were compared. The Fisher's exact test was applied to compare proportions, which avoids any violations of normal assumptions due to smaller sample sizes. Less conservative mid-*p*-values were estimated, and 47 RLGS loci with *p* < 0.06 were used for further analysis.

As methylation events are represented by binary variables, hierarchical cluster analysis of patient samples was performed by applying Jaccard noninvariant coefficient similarity metric [27], using the 47 RLGS loci with *p* < 0.06. Cluster analysis was performed three times, once with the initial group of 25 patients that was used to identify differential DNA methylation between adenocarcinomas and SCCs, then with a set of 15 new patients to validate the first result, and finally with both sample sets combined.

Real-time PCR data were analyzed by applying one-way ANOVA analysis followed by Scheffe test for multiple comparisons. Comparisons with *p* < 0.025 (97.5% CI) were considered significant.

Kruskal-Wallis rank sum tests and Fisher's exact tests were used to compare differences in baseline characteristics. Univariate and multivariate regression analyses were performed using the Cox proportional hazard regression model to determine the effects of various prognostic variables. Age was used as a dichotomous variable based on the median age value of the patients in the sample sets. OLIG1 index was used as a continuous variable composed of nine discrete values (1–9). In the multivariate model, the assumption of proportional hazards was examined for each variable by testing the significance of correlation coefficient between transformed survival time and the Schoenfeld residuals of that variable. All statistical analyses were performed using Splus and R (version 2.0.1) (http://www.r-project.org) softwares.

## Results

### Genome-Wide DNA Methylation Analysis of Human Adenocarcinomas and SCCs of the Lung

RLGS was performed on 11 adenocarcinomas (Adenos 1–11) and 14 SCCs (SCCs 1–14) to determine if these two lung tumor subtypes could be differentiated based on their aberrant DNA methylation patterns. The samples were selected so that gender, race, age range, and tumor differentiation were comparable in both groups. RLGS was performed using both NotI and AscI as restriction landmark enzymes. As previously reported [10], the recognition sequences of these enzymes occur preferentially within CpG islands as defined by Gardiner-Garden and Frommer [28], effectively creating a bias towards the assessment of DNA methylation in promoter sequences [15]. Additionally, recent bioinformatics analyses indicate that 92.7% of NotI sites fall within the 5′ end, inside, or 3′ end of transcripts (R.V. Davuluri, personal communication). The DNA methylation profile from each tumor was scored against a profile generated from tumor-free lung from the same patient. On average, the methylation status of 3,442 RLGS loci (range: 2,590–4,108) was analyzed per sample. The variation in the number of RLGS fragments analyzed per sample stemmed from individual differences in the quality of RLGS gels. Low level DNA degradation in specific samples resulted in RLGS fragments located in the periphery of the gel to become diffuse or not separated well enough to be analyzed accurately in all specimens. Aberrant DNA methylation was detected at least once in 395 of the total 4,108 different RLGS loci scored. The average frequency of CpG island methylation in the adenocarcinomas was 4.82% (range: 3.39%–6.26%) and 4.23% (range: 3.13%–5.42%) in the SCCs. The methylation level for each sample was calculated based on the exact number of RLGS loci scored for that sample.

**Figure 1:**
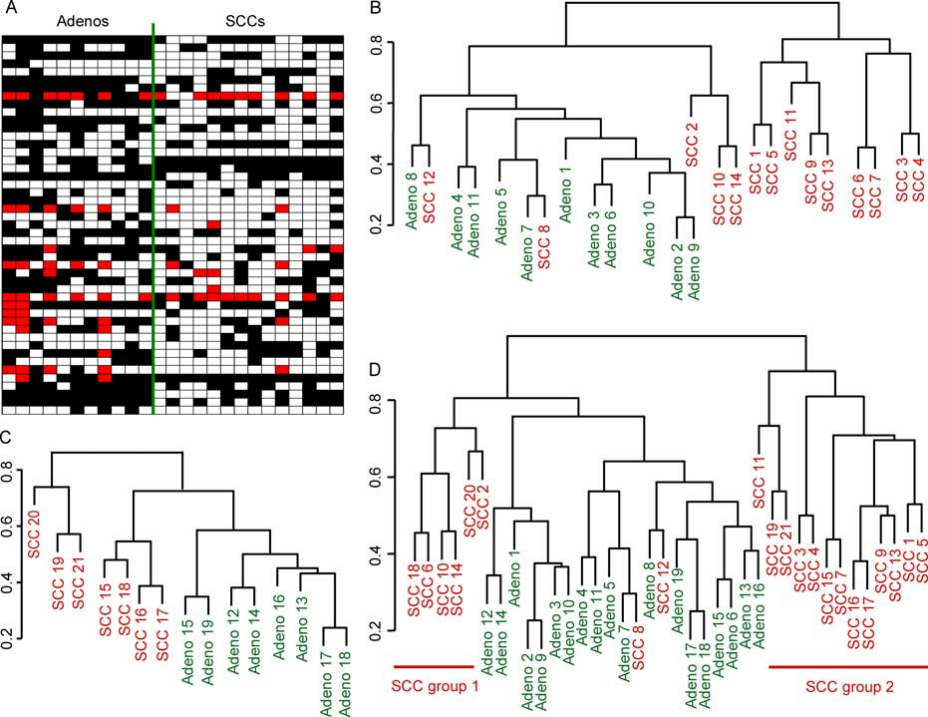
Aberrant DNA Methylation Profile and Cluster Analysis of Adenocarcinomas and SCCs of the Lung (A) DNA methylation patterns of the 47 RLGS fragments that distinguish adenocarcinomas from SCCs as recorded for the 25 primary tumor samples used to generate the cluster shown in (B). The adenocarcinoma and SCC sets are separated by a green line. Black boxes indicate DNA methylation; white boxes indicate absence of DNA methylation; red boxes indicate that the DNA methylation status of that RLGS fragment could not be determined. Each column represents a sample; each row represents an RLGS fragment. (B–C) Hierarchical clustering of adenocarcinoma and SCC samples. (B) Cluster comprising 25 samples (11 adenocarcinomas, 14 SCCs), based on 47 DNA methylation events. (C) Cluster comprising 15 samples (eight adenocarcinomas and seven SCCs), based on the DNA methylation information of the same 47 sequences as cluster (B). (D) Combined cluster from samples shown in clusters (B) and (C). SCCs groups 1 and 2 are underlined.

We identified 36 RLGS loci, whose methylation frequency was significantly different (*p* ≤ 0.050, Fisher's exact test) between the adenocarcinomas and the SCCs in the study. Of these, eight (22%) were methylated in only one of the tumor subtypes and not the other. The remaining sequences were methylated in both subtypes, but in varying frequencies (Figure 1A).

Next, hierarchical clustering was performed to determine if the aberrant methylation events detected in our RLGS scan were sufficient to distinguish the adenocarcinomas from the SCCs (Figure 1B). The best segregation of the tumors according to their subtype with the lowest number of misclassifications was achieved when the DNA methylation status of 47 RLGS loci was considered. While the adenocarcinomas clustered into one major group, the SCCs were split into two groups, one of them branching closer to the adenocarcinomas (SCCs 2, 10, and 14). Also, SCC8 and SCC12 clustered within the adenocarcinoma group. In order to validate if the DNA methylation status of these 47 RLGS loci could be applied to distinguish a new set of adenocarcinomas from a new set of SCCs, RLGS was performed on 15 additional samples (Adenos 12–19 and SCCs 15–21). These samples were also selected to ensure that gender, race, age range, and tumor differentiation were comparable in both tumor subtypes. Hierarchical clustering of these 15 samples showed a pattern where, again, the adenocarcinomas separated in one major group, while the SCCs were split into two groups (Figure 1C); a segregation pattern also seen in the combined cluster (Figure 1D). Interestingly, most of the SCCs grouping close to the adenocarcinomas (SCC group 1) were moderately differentiated (four of six), while the SCCs clustering entirely separately from the adenocarcinomas (SCC group 2) were predominantly poorly differentiated (eight of 13). This distribution, though not statistically significant, could indicate a trend that the two aberrant DNA methylation patterns observed in SCCs may reflect, in part, the differentiation state of the tumor.

### Differentially Methylated Loci in Adenocarcinomas and SCCs

**Figure 2:**
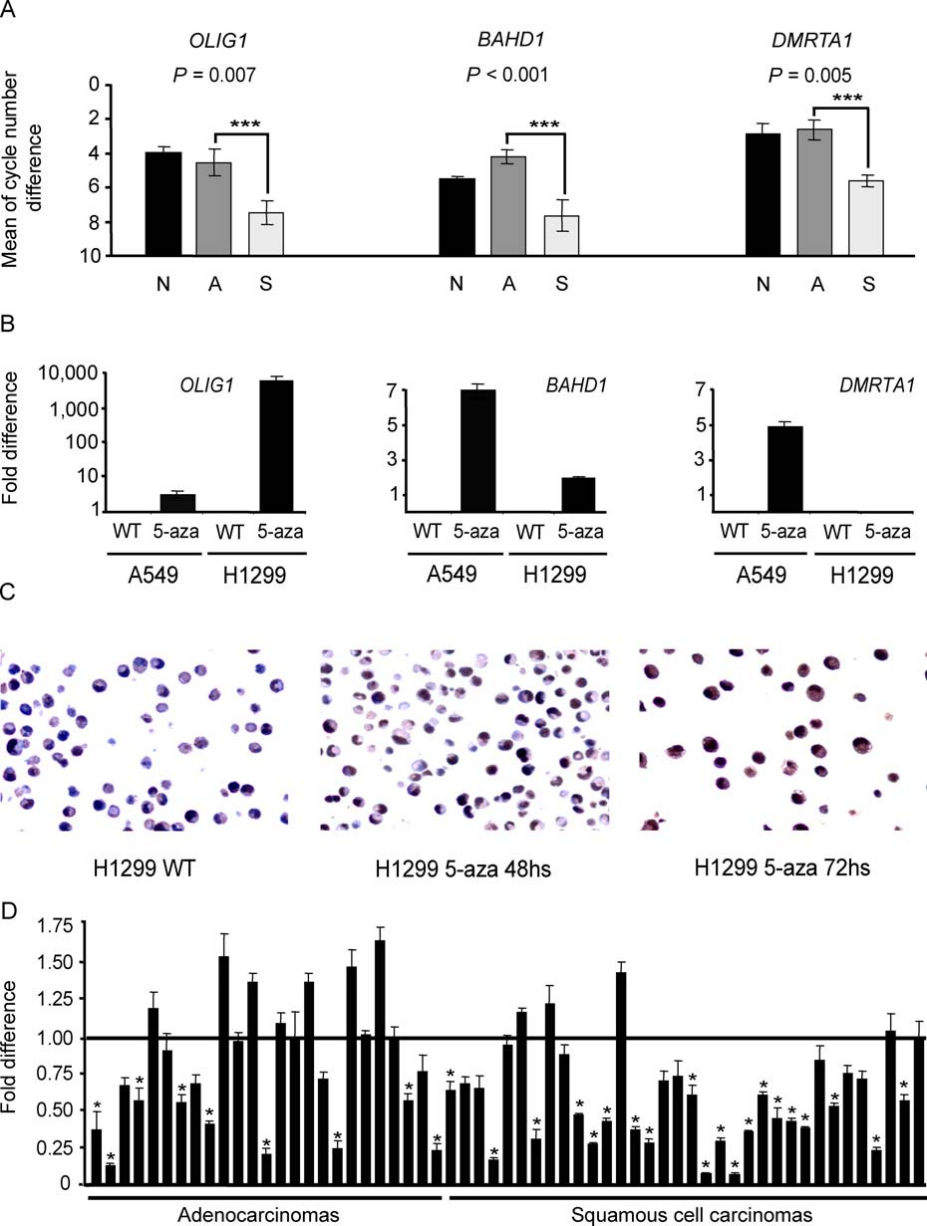
Real-Time PCR Analysis of Three Differentially Methylated Genes in Adenocarcinomas, SCCs, and Lung Cancer Cell Lines *OLIG1* immunohistochemistry in H1299 cells and *OLIG1* deletion analysis. (A) Real-time PCR data for *OLIG1*, *BAHD1,* and *DMRTA1* normalized to the expression of *CAMKK2*. The error bars indicate the standard deviation of nine different measurements per gene. The value for normal lung was established by taking the average mRNA expression of three different samples so as to try to capture the potential variability in the normal expression of each of the genes. ***; significant at the 97.5% confidence level; N, normal lung; A, adenocarcinoma; S, SCC. (B) *OLIG1, BAHD1,* and *DMRTA1* mRNA expression in A549 and H1299 cell lines treated with 1 μM 5-aza-dC for 72 h. The error bars indicate the standard deviation of nine individual measurements. (C) *OLIG1* immunohistochemistry on untreated and 1 μM 5-aza-dC treated H1299 cells. Treatment times are indicated underneath each panel. Photographs were taken at 400× magnification. (D) *OLIG1* DNA level for adenocarcinomas and SCCs. Each bar represents one sample. The error bars indicate the standard deviation of three independent measurements. Samples for which the level of *OLIG1* DNA was significantly lower than that of its matching tumor-free lung DNA (*p* < 0.050, one-tail Student's t-test) are indicated with a star.

Altogether, 33 of the 47 RLGS loci derived from our analysis were cloned either previously or in this study [13,16]. Of those 33 sequences, 28 were associated with a CpG island, and 26 matched an annotated gene locus (Table 1). Notably, many of the identified loci resided in chromosomal bands where loss of heterozygosity (LOH) had previously been described in lung cancer and/or other neoplasias [29]. To prioritize the experimental evaluation of the identified genes, SYBR green real-time PCR was performed on a new set of 12 adenocarcinomas (Adenos 20–31) and 12 SCCs (SCCs 22–33). The assay was carried out on 13 genes, those with the highest degree of differential DNA methylation between the two tumor subtypes. The real-time PCR results highlighted that of these 13 genes, *BAHD1, DMRTA1,* and *OLIG1* had the highest differential mRNA levels between adenocarcinomas and SCCs (*p* < 0.025, analysis of variance followed by Scheffe) (Figure 2A).

**Table 1:**
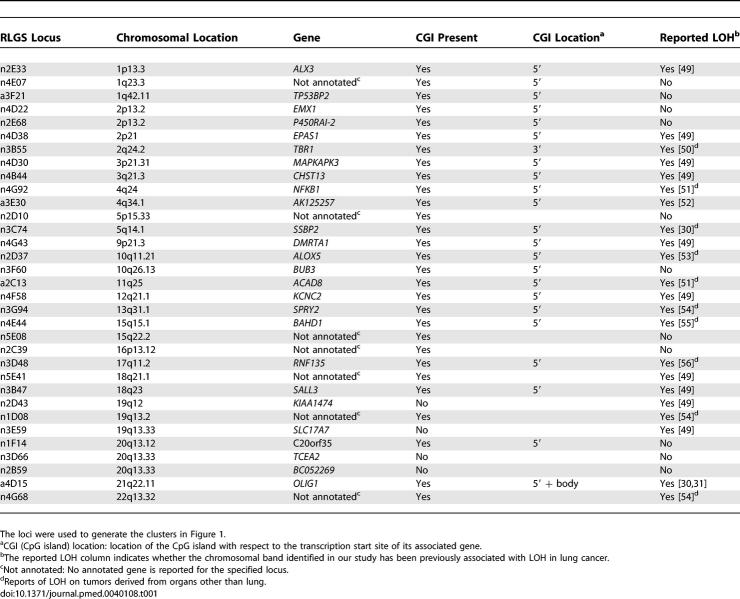
Chromosomal Location and Associated Genes for the 33 out of 47 Cloned RLGS Loci

Next, the human lung cancer cell lines A549 and H1299, in which *OLIG1*, *BAHD1,* and *DMRTA1* are methylated and not expressed, were treated with 1 μM of 5-aza-2′-deoxy-cytidine (5-aza-dC) for 48 and 72 h. The mRNA levels of all genes were up-regulated in at least one of the cell lines by 5-aza-dC (Figure 2B). *OLIG1* bisulfite DNA sequencing for both wild-type cell lines is shown in Figure S1. To confirm these results, OLIG1 immunohistochemistry was performed on the H1299 cells. As expected, OLIG1 protein expression was up-regulated upon treatment with the DNA demethylating agent (Figure 2C). Due to the lack of commercial antibodies for *BAHD1* and *DMRTA1,* COBRA was performed on both genes (Adenos 20–29 and SCCs 23–33). Our results showed that partial DNA methylation for *BAHD1* was detected in 90% of the samples, while partial DNA methylation for DMRTA1 was observed in 52% of them (unpublished data). These observations indicate that expression of *BAHD1*, *DMRTA1,* and *OLIG1* is directly or indirectly regulated by DNA methylation.

### *OLIG1* in Human Lung Cancer

Our DNA methylation, mRNA expression and 5-aza-dC reactivation data, coupled with literature describing recurrent LOH at chromosome 21q22.1 in SCCs of the lung [30,31], led us to select *OLIG1* for further study. Frequent LOH at microsatellite marker D21S12070 (43.8%) located 2.74 Mb upstream and marker D21S1445 (39.3%) located 0.93 Mb downstream of *OLIG1* was described in two reports [30,31]. Given the large distance between the two microsatellite markers, we tested the frequency of *OLIG1* deletions by directly assessing the presence of the *OLIG1* gene sequence in a subset of primary tumors. The assay was performed via quantitative real-time PCR on 25 adenocarcinomas (Adenos 20–44) and 34 SCCs (SCCs 22–55). We found that that 36% (*n* = 9) of the adenocarcinomas and 59% (*n* = 20) of the SCCs showed loss of *OLIG1* DNA compared to tumor-free lung, and the frequency of deletion was significantly higher in SCCs (*p* = 0.042, one-tail Z-test) (Figure 2D). This result is in agreement with previously published studies reporting significantly higher rates of LOH in SCCs than in adenocarcinomas [30,31]. The DNA methylation data generated by RLGS showed the same trend, with the frequency of *OLIG1* DNA methylation being significantly higher in SCCs.

**Figure 3:**
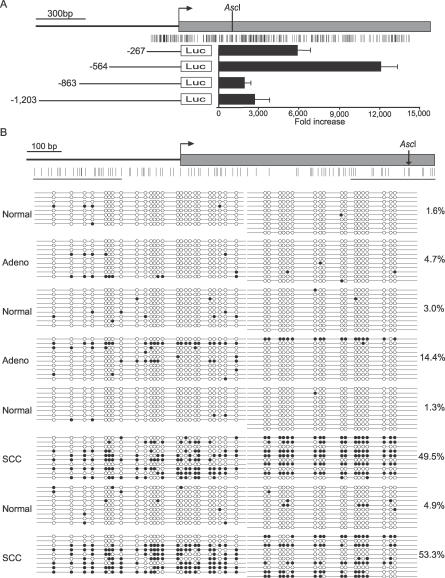
OLIG1 Luciferase Promoter Assay and Bisulfite DNA Sequencing (A) *OLIG1* gene diagram and luciferase activity determined for four deletion constructs in A549 cells. The gene is represented by a gray box with an arrow indicating the transcription start site. The location of the AscI site is indicated. The E2F3a promoter was used as a positive control for luciferase activity. The error bars indicate the standard deviation of three independent triplicate transfections. The gene diagram and constructs are drawn up to scale. (B) Bisulfite DNA sequencing of *OLIG1* in two adenocarcinomas, two SCCs, and four tumor-free lung samples derived from the same patients. The gene diagram on top indicates the relative location of the sequenced products in relation to the CpG sites within the CpG island (vertical lines), the transcription start site (horizontal arrow), and the exon (gray box). A total of eight to ten clones were sequenced per sample. Each line represents an individual clone and each circle represents a CpG dinucleotide. Solid circle, methylated cytosines; open circle, unmethylated cytosines; vertical arrow, AscI site.

To determine the location of the *OLIG1* promoter, we generated four luciferase constructs (Figure 3A). The constructs were transfected individually into A549 cells and assayed for luciferase activity. Our results showed that the region 267 bp upstream of the *OLIG1* transcription start site was sufficient to drive luciferase expression, and that a putative enhancer element might be located between −267 bp and −566 bp, because of the significantly higher luciferase activity of the longer construct (*p* < 0.001, analysis of variance) (Figure 3A). Thus, we focused on the 560-bp region upstream of *OLIG1* for further DNA methylation analysis.

Bisulfite DNA sequencing was performed on eight human lung samples (two adenocarcinomas, two SCCs, and their matching tumor-free lung tissues). A 260-bp PCR product spanning from −391 bp to −131 bp containing 25 CpG dinucleotides was generated. Another 203-bp PCR product containing 18 CpG dinucleotides was produced to cover the region from +296bp to +499 bp, where the AscI site (landmark enzyme in RLGS) is located. In both regions tested, the levels of DNA methylation were significantly higher in SCCs than in adenocarcinomas (*p* < 0.001, one-tail Z-test) (Figure 3B).

**Figure 4:**
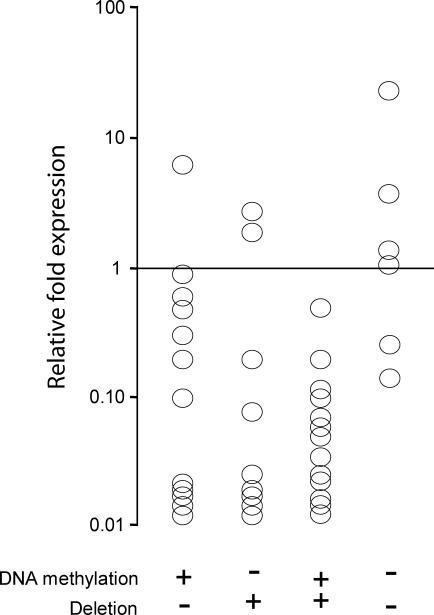
*OLIG1* mRNA Expression in Primary Tumor Samples in Relation to *OLIG1* DNA Methylation Levels and Deletions at the OLIG1 Locus Each circle represents a single sample. The presence of DNA methylation and/or deletions is indicated at the bottom of each sample column. mRNA expression levels are indicated in relation to normal lung, which was arbitrarily set as 1.

In order to establish a correlation between *OLIG1* DNA methylation, frequency of deletions at the *OLIG1* locus and *OLIG1* mRNA expression, Bio-COBRA, a technique that allows for the rapid and accurate quantification of DNA methylation in a sensitive and reproducible manner [19,21], was performed on a subset (41 out of 59) of the samples utilized to generate the *OLIG1* deletion data already described. The DNA methylation status of four BstUI sites was measured in a 260-bp PCR product extending from −391bp to −131bp of the *OLIG1* locus. DNA methylation was detected in 26 samples, ranging from 7.0% to 100% (mean 54.9%). These DNA methylation values were then combined with mRNA expression and deletion data. In 11 out of 13 samples in which DNA methylation alone was detected, reduced mRNA expression levels were shown compared to normal lung, as also was shown in seven out of nine samples in which *OLIG1* deletions alone were detected. All 13 samples in which concomitant *OLIG1* DNA methylation and *OLIG1* deletions were detected showed reduced mRNA levels, while two out of six of the samples in which no DNA methylation or deletions were assessed showed a reduction in *OLIG1* mRNA expression (Figure 4). Taken together, these data indicate that DNA methylation and deletions at the *OLIG1* locus in primary human lung tumors can be correlated with a reduction in *OLIG1* at the mRNA level.

### OLIG1 Immunohistochemistry on Lung Tissue Arrays

**Figure 5:**
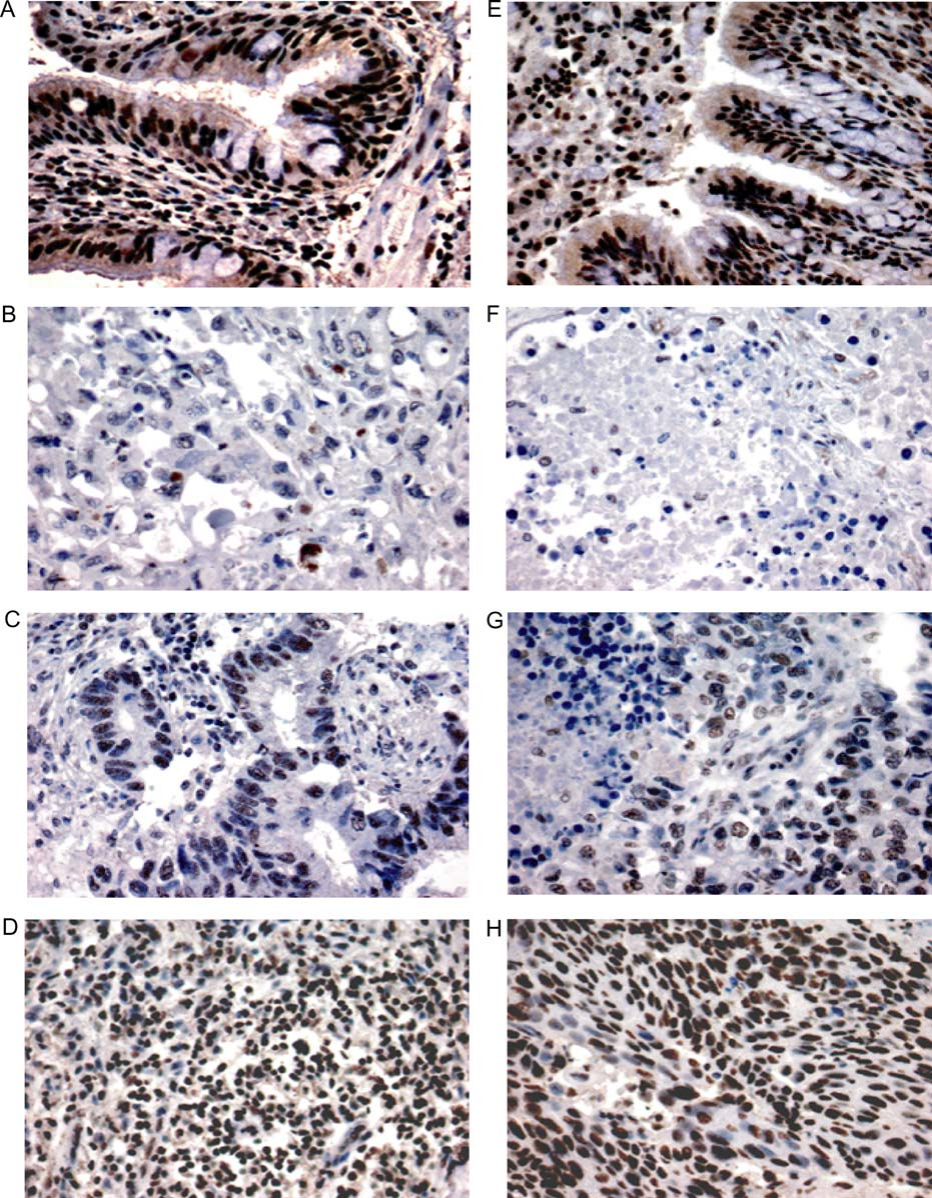
*OLIG1* Immunohistochemistry on a Lung Tissue Array *OLIG1* immunohistochemistry on (A and E) tumor-free lung, (B) an OLIG1 negative adenocarcinoma, and (F) an *OLIG1* negative SCC; (C) a low *OLIG1* expressing adenocarcinoma and (G) a low *OLIG1* expressing SCC are shown; (D) a high *OLIG1* expressing adenocarcinoma and (H) a high *OLIG1* expressing SCC are shown. All images were acquired at 400× magnification.

OLIG1 immunohistochemistry was performed on TMA1 comprising 59 adenocarcinomas (Adenos 45–103), 74 SCCs (SCCs 56–129), six tumor-free lung, and four human brain specimens. The immunohistochemical results were scored and an OLIG1 index value was assigned to each sample. The index values ranged from one (no expression) to nine (normal expression). Positive staining was detected in nuclei, indicating the correct localization of the target protein (Figure 5A–5H). Our analysis determined that 78% (*n* = 46) of adenocarcinomas and 58% (*n* = 42) of SCC were either negative or expressed OLIG1 protein at low levels (Table S5). In light of the high number of OLIG1 negative and low expressing cases in both lung tumor subtypes, we hypothesized that OLIG1 protein expression may influence survival in NSCLC patients.

To test this hypothesis, univariate and multivariate analyses were performed. All clinical and geographical variables available for the dataset (gender, age, tumor subtype, and T and N stages) were included in the models in order to account for potentially confounding factors independent of OLIG1 index, which may affect survival. The results of these analyses yielded a hazard ratio of 0.86 for OLIG1 index (95% CI 0.76–0.98, *p* = 0.023), indicating an association between reduced OLIG1 protein expression and reduced overall survival. In our analysis the OLIG1 index variable was composed of nine discrete values (1–9), where 1 represents lack of protein expression and 9 represents normal protein levels, as described in the Methods section. Therefore, our results indicate that for every unit increase in OLIG1 index, there is a risk reduction of 14% in relation to the risk associated with the lower index. For example, an OLIG1 index of 6 is associated with a 14% reduction in the risk afforded by an OLIG1 index of 5. By the same token, an OLIG1 index of 5 is associated with a 14% decrease in the risk afforded by an OLIG1 index of 4.

In order to validate our observations, OLIG1 immunohistochemistry was performed on an independent sample set (TMA2), comprising 74 adenocarcinomas (Adenos 104–182) and 79 SCCs (SCCs 130–208). The tissue cores were scored as previously described, and an OLIG1 index value was assigned to each sample. After completion of the data collection, univariate and multivariate analyses were performed on the dataset. The analyses were carried out in the same manner as for TMA1, including gender, age, tumor subtype, and T and N stage variables in the models. For this second dataset, the OLIG1 index hazard ratio was assessed at 0.83 (95% CI 0.74–0.93, *p* = 0.0012) lending further support to the observation that reduced OLIG1 protein expression is associated with reduced overall survival.

In an effort to improve the precision of the multivariate model, TMA1 and TMA2 were combined and reanalyzed in the same fashion as each individual dataset. The rationale for this approach was to increase the sample number, thereby increasing the statistical power and, potentially, the accuracy of the analysis. The OLIG1 index hazard ratio for the combined data was determined at 0.84 (95% CI 0.77–0.91 and *p* < 0.001). The complete Cox proportional hazard model for TMA1 and TMA2 combined is shown in Table 2.

**Table 2:**
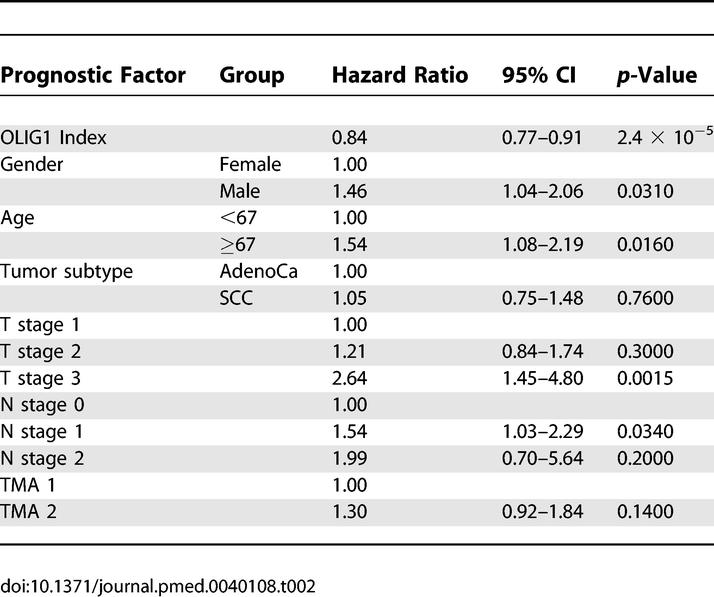
Multivariate Analysis of TMAs 1 and 2 Combined (*n* = 285)

Finally, we calculated the OLIG1 index hazard ratio for patients who were positive and negative for OLIG1 protein expression. This hazard ratio was generated by dividing the combined sample sets (*n* = 285) into two groups. Samples with an OLIG1 index ≤3 were considered negative, while samples with an index ≥4 were considered positive [25]. The hazard ratio for this calculation was 0.54 (95% CI 0.38–0.761 and *p* < 0.001), indicating a 54% lower risk for OLIG1-positive cases. From this multivariate model, the probability of survival at five years was calculated for both groups. For OLIG1-positive cases, the probability of survival at five years was assessed at 0.62 (95% CI 0.55–0.70), while for OLIG1-negative cases the probability of survival at five years was determined at 0.38 (95% CI 0.24–0.50). The difference between both survival probabilities, 0.24, was statistically significant (95% CI 0.11–0.36), further strengthening our previous observations.

Overall, the comprehensive statistical analysis of our datasets led us to conclude that reduced OLIG1 protein expression is associated with reduced overall survival, and this association is independent of clinical variables such as tumor subtype, T and N stages, or geographical variables such as gender and age. In particular we were able to show that survival at 60 months, a common clinical parameter for assessing lung cancer prognosis, is significantly associated with OLIG1 protein expression.

## Discussion

In this study we have demonstrated that lung adenocarcinomas and SCCs can be distinguished by the DNA methylation status of 47 discrete loci. This is a remarkable observation, since it not only lends further support to the fact that aberrant CpG island methylation is nonrandom [10], but it also indicates that different subtypes of neoplasias arising from the same organ can potentially be distinguished by their aberrant DNA methylation patterns.

One of the 47 aberrantly methylated loci was *OLIG1,* a basic helix-loop-helix transcription factor required for oligodendrocyte differentiation but of unknown function in adult lung [32]. Immunohistochemical analysis of a large set of adenocarcinomas and SCCs uncovered missing or reduced OLIG1 protein expression in 68% of the specimens tested, suggesting that abrogation of OLIG1 might be of clinical relevance in these subtypes of NSCLC. The impact of OLIG1 protein expression on patient survival was assessed by univariate and multivariate analyses. Cox proportional hazard models indicated that lack of OLIG1 protein was strongly associated with poor survival in NSCLC patients. Validation of these observations in an independent dataset mirrored the results first generated, further strengthening this association. Altogether, our results suggest that OLIG1 protein expression may provide an additional clinically useful parameter to determine the utility of supplementary therapy for patients suffering from lung NSCLC, especially since survival at 60 months is significantly correlated with OLIG1 protein expression. This finding is potentially of great significance, as the addition of postoperative adjuvant chemotherapy in T2N0 NSCLC, for example, is currently a matter of great debate [33,34].

The percentage of samples lacking OLIG1 protein was higher than expected within the adenocarcinoma subgroup. On the basis of the totality of the data collected in this study, it is possible that a post-transcriptional mechanism acting preferentially in the adenocarcinomas may account for either lack of *OLIG1* mRNA translation or rapid degradation of the OLIG1 protein product. This scenario reconciles the initial observations of lower DNA methylation and higher mRNA expression in adenocarcinomas compared to SCCs with the later finding of a higher proportion of *OLIG1* negative adenocarcinomas. Nevertheless, this phenomenon deserves further investigation. The corroboration of a tumor subtype-specific post-translational regulatory mechanism in lung cancer would be an immense contribution toward further understanding the etiology of this disease.

The importance of *OLIG1* expression in adult lung may be explained, in part, by extrapolation of known functions of this gene in oligodendrocyte development [35,36]. It has been shown that oligodendrocytes derived from *OLIG1^−^* ^/*−*^ mice are unable to differentiate [36], suggesting that at least one of the functions of *OLIG1* may pertain to initiation or maintenance of cellular differentiation. At the same time, *sonic hedgehog (SHH),* a secreted ligand of the hedgehog-signaling pathway known to be overexpressed in lung cancer [37], has been shown to be necessary and sufficient to activate *OLIG* genes in oligodendrocytes [38]. Thus, abrogation of OLIG1 protein expression may play a role in inhibiting cellular differentiation, but it could also contribute to the tumor phenotype in other ways through some of its downstream targets. *MAG*, a single-pass type I transmembrane protein involved in brain cellular adhesion [39,40] is highly expressed in adult lung [41], and it is also a known target of *OLIG1* [39]. In light of these genetic interactions, a growth advantage could be conferred to tumor cells that overexpress SHH through interaction with currently unknown growth promoting targets, while at the same time abrogating OLIG1 expression concomitant to MAG down-regulation. This scenario would explain the high frequency of deletions and DNA methylation observed at the *OLIG1* locus. This hypothesis is reinforced by our observation that N0 NSCLC cases are more likely to be OLIG1 positive than N1 cases. Therefore, this phenotypic difference could stem, in part, from lack or reduced *MAG* expression in OLIG1 negative tumors, which could facilitate detachment of tumor cells from the primary tumor mass.

Taking into consideration the relationship between DNA methylation and gene expression, our study demonstrates that genome-wide DNA methylation patterns can be as useful in tumor subtype distinction as gene expression profiling, an approach that has been successfully utilized in the past to distinguish not only lung tumor subtypes but also phenotypic differences associated with survival within a lung tumor subclass [42–44]. In light of our results, the establishment of differential DNA methylation patterns could reflect an intrinsic difference in the cellular origin [45] of each of the tumor subtypes, or by distinct oncogenic pathways activated predominantly in one subtype over the other. It has been well documented that gains in 3q22–q26 where the alpha catalytic subunit of phosphatidylinositol 3-kinase is located, occurs almost exclusively in SCCs [46]. Overexpression of this gene could be correlated with increased activity of its downstream effector, protein kinase B, in this lung tumor subtype [46]. Given the evidence that DNA methylation may be the result of a priori down-regulation of gene expression [47,48], the establishment of differential DNA methylation patterns between lung tumor subtypes may be the result of distinct oncogenic activities affecting primarily one type of neoplasia and not the other. Additional studies will be needed to fully elucidate the mechanisms governing the establishment of tumor subtype specific DNA methylation patterns.

## Supporting Information

Alternative Language Abstract S1 Translation of the abstract into Spanish by Romulo Martin Brena. (21 KB DOC)

Figure S1 *Olig1* Bisulfite DNA Sequencing in A549 and H1299 Cell Lines (459 KB TIF)

Table S1 Adenocarcinoma and SCC Samples Analyzed by RLGS to Generate Clusters All clusters are shown in Figure 1.
(A) Adenocarcinoma samples; samples A1 to A11 were used to generate cluster 1B. Samples A12 to A19 were used to generate cluster 1C. All samples are present in cluster 1D.
(B) Summary of the clinical and demographic characteristics for the samples listed in part A; the age range is indicated in parenthesis.
(C) SCC samples; samples S1 to S14 were used to generate cluster 1B. Samples S15 to S21 were used to generate cluster 1C. All samples are present in cluster 1D.
(D) Summary of the clinical and demographic characteristics for the samples listed in part C. The age range is indicated in parenthesis.
(107 KB DOC)

Table S2 Clinical Characteristics for Tumor Samples Present in TMA1 That Met All the Quality Control Criteria to Be Included in the Analysis for OLIG1 Protein Expression (A) Adenocarcinoma samples; the age range for the sample set is indicated in brackets underneath the mean age value.
(B) SCC samples; the age range for the sample set is indicated in brackets underneath the mean age value.
(62 KB DOC)

Table S3 Clinical Characteristics for Tumor Samples Present in TMA2 That Met All the Quality Control Criteria to Be Included in the Analysis for OLIG1 Protein Expression (A) Adenocarcinoma samples; the age range for the sample set is indicated in brackets underneath the mean age value.
(B) SCC samples; the age range for the sample set is indicated in brackets underneath the mean age value.
(38 KB DOC)

Table S4 Average OLIG1 Index for Each of the Samples Used for Immunohistochemistry in TMA1 Samples are classified into three categories: positive (index > 6), negative (index < 4), and low level (index 4–5).
(39 KB DOC)

Table S5 Primer Sequences and Conditions for All PCR Reactions Performed in This Study (A) Primer sequences and PCR conditions used to evaluate mRNA expression in the genes listed on the left. The *OLIG1* and *CAMKK2* primers were also used to assess for *OLIG1* deletions in primary tumors.
(B) Primer sequences with their corresponding annealing temperatures used to amplify the *BAHD1* and *DMRTA1* for Bio-COBRA.
(C) Primer sequences with their corresponding annealing temperatures used to amplify the *OLIG1* constructs used in the luciferase assays.
(D) Primer sequences with their corresponding annealing temperatures used to amplify the *OLIG1* regions selected for bisulfite DNA sequencing.
(146 KB DOC)

### Accession Numbers

Accession numbers for all genes analyzed or mentioned in this study can be found at UniGene (http://www.ncbi.nlm.nih.gov/entrez/query.fcgi?db = unigene).

## Abbreviations

COBRA: combined bisulfite restriction analysis
LOH: loss of heterozygosity
NSCLC: non-small cell lung cancer
RLGS: restriction landmark genomic scanning
SCC: squamous cell carcinoma
TMA: tissue microarray
TNM: tumor, node, metastasis

## References

[pmed-0040108-b001] Jemal A, Murray T, Ward E, Samuels A, Tiwari RC (2005). Cancer statistics, 2005. CA Cancer J Clin.

[pmed-0040108-b002] Parkin DM, Bray F, Ferlay J, Pisani P (2001). Estimating the world cancer burden: Globocan 2000. Int J Cancer.

[pmed-0040108-b003] Jemal A, Clegg LX, Ward E, Ries LA, Wu X (2004). Annual report to the nation on the status of cancer, 1975–2001, with a special feature regarding survival. Cancer.

[pmed-0040108-b004] Greene FL, Sobin LH (2002). The TNM system: Our language for cancer care. J Surg Oncol.

[pmed-0040108-b005] Vielh P, Spano JP, Grenier J, Le Chevalier T, Soria JC (2005). Molecular prognostic factors in resectable non-small cell lung cancer. Crit Rev Oncol Hematol.

[pmed-0040108-b006] Potti A, Mukherjee S, Petersen R, Dressman HK, Bild A (2006). A genomic strategy to refine prognosis in early-stage non-small-cell lung cancer. N Engl J Med.

[pmed-0040108-b007] Jones PA, Baylin SB (2002). The fundamental role of epigenetic events in cancer. Nat Rev Genet.

[pmed-0040108-b008] Baylin SB, Herman JG, Graff JR, Vertino PM, Issa JP (1998). Alterations in DNA methylation: A fundamental aspect of neoplasia. Adv Cancer Res.

[pmed-0040108-b009] Esteller M, Corn PG, Baylin SB, Herman JG (2001). A gene hypermethylation profile of human cancer. Cancer Res.

[pmed-0040108-b010] Costello JF, Fruhwald MC, Smiraglia DJ, Rush LJ, Robertson GP (2000). Aberrant CpG-island methylation has non-random and tumour-type-specific patterns. Nat Genet.

[pmed-0040108-b011] Esteller M (2003). Cancer epigenetics: DNA methylation and chromatin alterations in human cancer. Adv Exp Med Biol.

[pmed-0040108-b012] Brena RM, Huang TH, Plass C (2006). Quantitative assessment of DNA methylation: Potential applications for disease diagnosis, classification, and prognosis in clinical settings. J Mol Med.

[pmed-0040108-b013] Dai Z, Lakshmanan RR, Zhu WG, Smiraglia DJ, Rush LJ (2001). Global methylation profiling of lung cancer identifies novel methylated genes. Neoplasia.

[pmed-0040108-b014] Richardson B (2003). Impact of aging on DNA methylation. Ageing Res Rev.

[pmed-0040108-b015] Dai Z, Weichenhan D, Wu YZ, Hall JL, Rush LJ (2002). An AscI boundary library for the studies of genetic and epigenetic alterations in CpG islands. Genome Res.

[pmed-0040108-b016] Zardo G, Tiirikainen MI, Hong C, Misra A, Feuerstein BG (2002). Integrated genomic and epigenomic analyses pinpoint biallelic gene inactivation in tumors. Nat Genet.

[pmed-0040108-b017] Tada Y, Brena RM, Hackanson B, Morrison C, Otterson GA (2006). Epigenetic modulation of tumor suppressor CCAAT/enhancer binding protein alpha activity in lung cancer. J Natl Cancer Inst.

[pmed-0040108-b018] Auer H, Lyianarachchi S, Newsom D, Klisovic MI, Marcucci G (2003). Chipping away at the chip bias: RNA degradation in microarray analysis. Nat Genet.

[pmed-0040108-b019] Brena RM, Auer H, Kornacker K, Hackanson B, Raval A (2006). Accurate quantification of DNA methylation using combined bisulfite restriction analysis coupled with the Agilent 2100 Bioanalyzer platform. Nucleic Acids Res.

[pmed-0040108-b020] Eads CA, Laird PW (2002). Combined bisulfite restriction analysis (COBRA). Methods Mol Biol.

[pmed-0040108-b021] Brena RM, Auer H, Kornacker K, Plass C (2006). Quantification of DNA methylation in electrofluidics chips (Bio-COBRA). Nat Protoc.

[pmed-0040108-b022] Clark SJ, Harrison J, Paul CL, Frommer M (1994). High sensitivity mapping of methylated cytosines. Nucleic Acids Res.

[pmed-0040108-b023] Abdel-Rahman MH, Yang Y, Zhou XP, Craig EL, Davidorf FH (2006). High frequency of submicroscopic hemizygous deletion is a major mechanism of loss of expression of PTEN in uveal melanoma. J Clin Oncol.

[pmed-0040108-b024] Goethals L, Perneel C, Debucquoy A, De Schutter H, Borghys D (2006). A new approach to the validation of tissue microarrays. J Pathol.

[pmed-0040108-b025] Allred DC, Harvey JM, Berardo M, Clark GM (1998). Prognostic and predictive factors in breast cancer by immunohistochemical analysis. Mod Pathol.

[pmed-0040108-b026] Poola I, DeWitty RL, Marshalleck JJ, Bhatnagar R, Abraham J (2005). Identification of MMP-1 as a putative breast cancer predictive marker by global gene expression analysis. Nat Med.

[pmed-0040108-b027] Kaufman L, Rousseeuw PJ (1990). Finding groups in data: An introduction to cluster analysis.

[pmed-0040108-b028] Gardiner-Garden M, Frommer M (1987). CpG islands in vertebrate genomes. J Mol Biol.

[pmed-0040108-b029] Breuer RH, Postmus PE, Smit EF (2005). Molecular pathology of non-small-cell lung cancer. Respiration.

[pmed-0040108-b030] Sato S, Nakamura Y, Tsuchiya E (1994). Difference of allelotype between squamous cell carcinoma and adenocarcinoma of the lung. Cancer Res.

[pmed-0040108-b031] Lee EB, Park TI, Park SH, Park JY (2003). Loss of heterozygosity on the long arm of chromosome 21 in non-small cell lung cancer. Ann Thorac Surg.

[pmed-0040108-b032] Zhou Q, Wang S, Anderson DJ (2000). Identification of a novel family of oligodendrocyte lineage-specific basic helix-loop-helix transcription factors. Neuron.

[pmed-0040108-b033] Winton T, Livingston R, Johnson D, Rigas J, Johnston M (2005). Vinorelbine plus cisplatin vs. observation in resected non-small-cell lung cancer. N Engl J Med.

[pmed-0040108-b034] Arriagada R, Bergman B, Dunant A, Le Chevalier T, Pignon JP (2004). Cisplatin-based adjuvant chemotherapy in patients with completely resected non-small-cell lung cancer. N Engl J Med.

[pmed-0040108-b035] Ligon KL, Fancy SP, Franklin RJ, Rowitch DH (2006). Olig gene function in CNS development and disease. Glia.

[pmed-0040108-b036] Arnett HA, Fancy SP, Alberta JA, Zhao C, Plant SR (2004). bHLH transcription factor Olig1 is required to repair demyelinated lesions in the CNS. Science.

[pmed-0040108-b037] Watkins DN, Berman DM, Burkholder SG, Wang B, Beachy PA (2003). Hedgehog signalling within airway epithelial progenitors and in small-cell lung cancer. Nature.

[pmed-0040108-b038] Alberta JA, Park SK, Mora J, Yuk D, Pawlitzky I (2001). Sonic hedgehog is required during an early phase of oligodendrocyte development in mammalian brain. Mol Cell Neurosci.

[pmed-0040108-b039] Xin M, Yue T, Ma Z, Wu FF, Gow A (2005). Myelinogenesis and axonal recognition by oligodendrocytes in brain are uncoupled in Olig1-null mice. J Neurosci.

[pmed-0040108-b040] Sow A, Lamant M, Bonny JM, Larvaron P, Piaud O (2006). Oligodendrocyte differentiation is increased in transferrin transgenic mice. J Neurosci Res.

[pmed-0040108-b041] Shmueli O, Horn-Saban S, Chalifa-Caspi V, Shmoish M, Ophir R (2003). GeneNote: Whole genome expression profiles in normal human tissues. C R Biol.

[pmed-0040108-b042] Yamagata N, Shyr Y, Yanagisawa K, Edgerton M, Dang TP (2003). A training-testing approach to the molecular classification of resected non-small cell lung cancer. Clin Cancer Res.

[pmed-0040108-b043] Beer DG, Kardia SL, Huang CC, Giordano TJ, Levin AM (2002). Gene-expression profiles predict survival of patients with lung adenocarcinoma. Nat Med.

[pmed-0040108-b044] Bhattacharjee A, Richards WG, Staunton J, Li C, Monti S (2001). Classification of human lung carcinomas by mRNA expression profiling reveals distinct adenocarcinoma subclasses. Proc Natl Acad Sci U S A.

[pmed-0040108-b045] Lee JS, Heo J, Libbrecht L, Chu IS, Kaposi-Novak P (2006). A novel prognostic subtype of human hepatocellular carcinoma derived from hepatic progenitor cells. Nat Med.

[pmed-0040108-b046] Massion PP, Kuo WL, Stokoe D, Olshen AB, Treseler PA (2002). Genomic copy number analysis of non-small cell lung cancer using array comparative genomic hybridization: Implications of the phosphatidylinositol 3-kinase pathway. Cancer Res.

[pmed-0040108-b047] Leu YW, Yan PS, Fan M, Jin VX, Liu JC (2004). Loss of estrogen receptor signaling triggers epigenetic silencing of downstream targets in breast cancer. Cancer Res.

[pmed-0040108-b048] Frigola J, Song J, Stirzaker C, Hinshelwood RA, Peinado MA (2006). Epigenetic remodeling in colorectal cancer results in coordinate gene suppression across an entire chromosome band. Nat Genet.

[pmed-0040108-b049] Virmani AK, Fong KM, Kodagoda D, McIntire D, Hung J (1998). Allelotyping demonstrates common and distinct patterns of chromosomal loss in human lung cancer types. Genes Chromosomes Cancer.

[pmed-0040108-b050] Aghmesheh M, Suo Z, Friedlander M, Nesland JM, Kaern J (2006). Chromosome 2q24.2 is lost in sporadic but not in BRCA1-associated ovarian carcinomas. Pathology.

[pmed-0040108-b051] Yuan E, Li CM, Yamashiro DJ, Kandel J, Thaker H (2005). Genomic profiling maps loss of heterozygosity and defines the timing and stage dependence of epigenetic and genetic events in Wilms' tumors. Mol Cancer Res.

[pmed-0040108-b052] Girard L, Zochbauer-Muller S, Virmani AK, Gazdar AF, Minna JD (2000). Genome-wide allelotyping of lung cancer identifies new regions of allelic loss, differences between small cell lung cancer and non-small cell lung cancer, and loci clustering. Cancer Res.

[pmed-0040108-b053] Dumur CI, Dechsukhum C, Ware JL, Cofield SS, Best AM (2003). Genome-wide detection of LOH in prostate cancer using human SNP microarray technology. Genomics.

[pmed-0040108-b054] Nowak NJ, Gaile D, Conroy JM, McQuaid D, Cowell J (2005). Genome-wide aberrations in pancreatic adenocarcinoma. Cancer Genet Cytogenet.

[pmed-0040108-b055] Kee HJ, Shin JH, Chang J, Chung KY, Shin DH (2003). Identification of tumor suppressor loci on the long arm of chromosome 15 in primary small cell lung cancer. Yonsei Med J.

[pmed-0040108-b056] Fukumoto M, Nakayama K (2006). Ovarian epithelial tumors of low malignant potential: Are they precursors of ovarian carcinoma?. Pathol Int.

