# Supplementary material for: Aberrant DNA Methylation of OLIG1, a Novel Prognostic Factor in Non-Small Cell Lung Cancer
Source: PLoS Med. 2007 Mar 27;4(3):e108. doi: 10.1371/journal.pmed.0040108 (PMC1831740; doi:10.1371/journal.pmed.0040108)
Supplement: Alternative Language Abstract S1 — (21 KB DOC) [file pmed.0040108.sd001.doc]

**Resumen**

**Antecedentes**

El cáncer de pulmón es la neoplasia que causa la mayor cantidad de muertes en el mundo. Actualmente, el pronóstico más preciso para pacientes con cánceres de células no pequeñas es logrado mediante la categorización de tumores por el sistema TNM. Sin embargo, la sobre vida de pacientes con tumores operables varía significativamente. Esto indica que es necesario considerar nuevos factores que puedan ayudar a mejorar el pronóstico de la enfermedad, en especial dentro de subgrupos TNM.

**Métodos y Resultados**

En este estudio, investigamos si los adenocarcinomas y carcinomas de células escamosas pulmonares pueden ser distinguidos en base a sus patrones globales de metilación de ADN. Con este objetivo, analizamos 40 tumores primarios mediante *restriction landmark genomic scanning (RLGS)* e identificamos 47 genes. El análisis conjunto de esos 47 genes es suficiente para distinguir correctamente estas dos subclases de tumores. La expresión a nivel proteico de uno de esos genes, *oligodendrocyte transcription factor 1 (OLIG1)*, mostró una correlación significativa con la sobre vida. Además, la proporción de riesgo para pacientes con tumores que no expresan esta proteína es significativamente mayor comparada con la de esos pacientes con tumores que sí la expresan, aunque sea a niveles bajos.

**Conclusiones**

Análisis de variables de nuestros resultados confirmaron que la expresión a nivel proteico de OLIG1, conjuntamente con los estadios T y N afecta significativamente la sobre vida en pacientes con cáncer de pulmón de células no pequeñas. Nuestro estudio determinó un riesgo relativo de 0.84 (intervalo de confianza al 95%: 0.77-0.91, *p* < 0.001). En conclusión, nuestros resultados sugieren que la expresión proteica de OLIG1 puede ser utilizada como un nuevo factor de pronóstico. Este nuevo parámetro tiene el potencial de poder ayudar a determinar qué pacientes pudieran obtener un mayor beneficio mediante la aplicación de regímenes de quimioterapias más agresivos.
