# Supplementary material for: Aberrant DNA Methylation of OLIG1, a Novel Prognostic Factor in Non-Small Cell Lung Cancer
Source: PLoS Med. 2007 Mar 27;4(3):e108. doi: 10.1371/journal.pmed.0040108 (PMC1831740; doi:10.1371/journal.pmed.0040108)
Supplement: Table S1 — All clusters are shown in Figure 1. (A) Adenocarcinoma samples; samples A1 to A11 were used to generate cluster 1B. Samples A12 to A19 were used to generate cluster 1C. All samples are present in cluster 1D. (B) Summary of the clinical and demographic characteristics for the samples listed in part A; the age range is indicated in parenthesis. (C) SCC samples; samples S1 to S14 were used to generate cluster 1B. Samples S15 to S21 were used to generate cluster 1C. All samples are present in cluster 1D. (D) Summary of the clinical and demographic characteristics for the samples listed in part C. The age range is indicated in parenthesis. (107 KB DOC) [file pmed.0040108.st001.doc]

Table S1

A) Characteristics of the adenocarcinoma samples used to generate the clusters in Fig 1. Samples

A1 to A11 were used to generate cluster 1B. Samples A12 to A19 were used to generate cluster

1C. All samples are present in cluster 1D.

| ***SAMPLE ID*** | ***Diagnosis*** | ***Tumor DNA*** | ***Differentiation*** | ***Gender*** | ***Race*** | ***Age*** |
| --- | --- | --- | --- | --- | --- | --- |
| **A 1** | **Adeno** | **90%** | **Well** | **F** | **Caucasian** | **68** |
| **A 2** | **Adeno** | **90%** | **Poor** | **F** | **Caucasian** | **80** |
| **A 3** | **Adeno** | **90%** | **Poor** | **M** | **Caucasian** | **73** |
| **A 4** | **Adeno** | **90%** | **Moderate** | **M** | **Caucasian** | **63** |
| **A 5** | **Adeno** | **81%** | **Poor** | **M** | **N/A** | **78** |
| **A 6** | **Adeno** | **80%** | **Poor** | **F** | **Caucasian** | **57** |
| **A 7** | **Adeno** | **90%** | **Well** | **F** | **Caucasian** | **80** |
| **A 8** | **Adeno** | **75%** | **Moderate** | **M** | **N/A** | **74** |
| **A 9** | **Adeno** | **90%** | **Moderate** | **F** | **Caucasian** | **68** |
| **A 10** | **Adeno** | **76%** | **Well** | **F** | **N/A** | **70** |
| **A 11** | **Adeno** | **80%** | **Moderate** | **F** | **Caucasian** | **77** |
| **A 12** | **Adeno** | **100%** | **Poor** | **M** | **N/A** | **60** |
| **A 13** | **Adeno** | **75%** | **Poor** | **F** | **Caucasian** | **82** |
| **A 14** | **Adeno** | **100%** | **Moderate** | **M** | **Caucasian** | **80** |
| **A 15** | **Adeno** | **77%** | **Moderate** | **F** | **Caucasian** | **74** |
| **A 16** | **Adeno** | **70%** | **Poor** | **M** | **Caucasian** | **71** |
| **A 17** | **Adeno** | **70%** | **Poor** | **M** | **Black** | **59** |
| **A 18** | **Adeno** | **70%** | **Poor** | **F** | **Black** | **68** |
| **A 19** | **Adeno** | **71%** | **Well** | **M** | **Black** | **49** |

B) Summary of the demographic and clinical features of the samples listed in part a. The age range is indicated in parenthesis.

| **Gender distribution** | | **Tumor differentiation** | | **Race** | | **Mean tumor DNA** | **Mean age** | |
| --- | --- | --- | --- | --- | --- | --- | --- | --- |
| **M** | **47%** | **Well** | **21%** | **Caucasian** | **73%** | **82%** | **70** | |
| **F** | **53%** | **Moderate** | **32%** | **Black** | **16%** |  | **(49-82)** |  |
|  | | **Poor 47%** | | **N/A 11%** | |  |  | |

C) Characteristics of the squamous cell carcinoma samples used to generate the clusters in Fig 1.

Samples S1 to S14 were used to generate cluster 1B. Samples S15 to S21 were used to generate

cluster 1C. All samples are present in cluster 1D.

| ***SAMPLE ID*** | ***Diagnosis*** | ***Tumor DNA*** | ***Differentiation*** | ***Gender*** | ***Race*** | ***Age*** |
| --- | --- | --- | --- | --- | --- | --- |
| **S 1** | **SCC** | **77%** | **Moderate** | **F** | **N/A** | **72** |
| **S 2** | **SCC** | **84%** | **Poor** | **M** | **Caucasian** | **71** |
| **S 3** | **SCC** | **90%** | **Poor** | **F** | **Caucasian** | **66** |
| **S 4** | **SCC** | **86%** | **Moderate** | **F** | **Caucasian** | **71** |
| **S 5** | **SCC** | **75%** | **Well** | **F** | **Caucasian** | **47** |
| **S 6** | **SCC** | **88%** | **Moderate** | **N/A** | **N/A** | **N/A** |
| **S 7** | **SCC** | **76%** | **Poor** | **M** | **Caucasian** | **62** |
| **S 8** | **SCC** | **85%** | **Poor** | **M** | **Caucasian** | **74** |
| **S 9** | **SCC** | **77%** | **Poor** | **F** | **N/A** | **83** |
| **S 10** | **SCC** | **86%** | **Moderate** | **F** | **Caucasian** | **75** |
| **S 11** | **SCC** | **82%** | **Poor** | **M** | **Caucasian** | **64** |
| **S 12** | **SCC** | **70%** | **Poor** | **F** | **Caucasian** | **70** |
| **S 13** | **SCC** | **78%** | **Poor** | **F** | **Caucasian** | **68** |
| **S 14** | **SCC** | **74%** | **Moderate** | **M** | **Black** | **65** |
| **S 15** | **SCC** | **80%** | **Poor** | **M** | **N/A** | **71** |
| **S 16** | **SCC** | **100%** | **Well** | **M** | **Black** | **51** |
| **S 17** | **SCC** | **80%** | **Poor** | **M** | **Black** | **69** |
| **S 18** | **SCC** | **80%** | **Moderate** | **M** | **Caucasian** | **67** |
| **S 19** | **SCC** | **75%** | **Moderate** | **M** | **Caucasian** | **52** |
| **S 20** | **SCC** | **90%** | **Well** | **F** | **Black** | **65** |
| **S 21** | **SCC** | **75%** | **Poor** | **M** | **Caucasian** | **62** |

D) Summary of the demographic and clinical features of the samples listed in part a. The age range is indicated in parenthesis.

| **Gender distribution** | | **Tumor differentiation** | | **Race** | | **Mean tumor DNA** | **Mean age** | |
| --- | --- | --- | --- | --- | --- | --- | --- | --- |
| **M** | **52%** | **Well** | **15%** | **Caucasian** | **62%** | **81%** | **66** | |
| **F** | **43%** | **Moderate** | **33%** | **Black** | **19%** |  | **(47-83)** |  |
| **N/A 5%** | | **Poor 52%** | | **N/A 19%** | |  |  | |
