# Supplementary material for: Aberrant DNA Methylation of OLIG1, a Novel Prognostic Factor in Non-Small Cell Lung Cancer
Source: PLoS Med. 2007 Mar 27;4(3):e108. doi: 10.1371/journal.pmed.0040108 (PMC1831740; doi:10.1371/journal.pmed.0040108)
Supplement: Table S2 — (A) Adenocarcinoma samples; the age range for the sample set is indicated in brackets underneath the mean age value. (B) SCC samples; the age range for the sample set is indicated in brackets underneath the mean age value. (62 KB DOC) [file pmed.0040108.st002.doc]

Table S2

A) Primer sequences and PCR conditions used to evaluate mRNA expression in the genes listed on the left. The *OLIG1* and *CAMKK2* primers were also used to assess for *OLIG1* deletions in primary tumors.

| ***Gene*** | ***Forward primer*** | ***Reverse Primer*** | ***Real-time PCR amplification conditions*** | ***Product size*** |
| --- | --- | --- | --- | --- |
| **CAMKK2** | **5’-CTCTTCCAGTGGGCAAAGAG-3’** | **5’-GTGTCAACAAGGGGCTCAAT-3’** | **95°C x 3', (96°C x 30", 60°C x 20") x 35 cycles** | **220 bp** |
| **OLIG1** | **5’-CCCCAAAAGTAGCGTAACCA-3’** | **5’-CCGGTACTCCTGCGTGTTA-3’** | **95°C x 3', (96°C x 30", 62°C x 30") x 35 cycles** | **163 bp** |
| **BAHD1** | **5’-GCCCTTGCAGAATGAAGTGT-3’** | **5’-GGGAACCAGTCGTGTCTTTC-3’** | **95°C x 3', (96°C x 30", 62°C x 30") x 35 cycles** | **166 bp** |
| **DMRTA1** | **5’-CTTGAGACAGGCCAGTGGTT-3’** | **5’-TTTGTTTCCCAATGACACCA-3’** | **95°C x 3', (96°C x 30", 60°C x 30") x 35 cycles** | **191 bp** |
| **MAPKAPK3** | **5’-CACAGAGAGGCTGACCATCA-3’** | **5’-AGGAGCCGGTTGTTAGAGGT-3’** | **95°C x 3', (96°C x 30", 62°C x 30") x 35 cycles** | **219 bp** |
| **SPRY2** | **5’-TTTGCATCGAGACTGGATTT-3’** | **5’-GAGCTCTGGCCTCCATCA-3’** | **95°C x 3', (96°C x 30", 60°C x 30") x 35 cycles** | **150 bp** |
| **TP53BP2** | **5’-GCATGACAATCATCCACAGG-3’** | **5’-TGTGGAAGTTTCAGGCCAAG-3’** | **95°C x 3', (96°C x 30", 62°C x 30") x 35 cycles** | **150 bp** |
| **NFkB1** | **5’-CACCAAGCAATTGAAGTGA-3’** | **5’-AGGTCCTTCCTGCCCATAAT-3’** | **95°C x 3', (96°C x 30", 62°C x 30") x 35 cycles** | **247 bp** |
| **P450RAI2** | **5’-GCTACCTGGACTGCGTCATC-3’** | **5’-CACGTTCACGTCTTTGAACA-3’** | **95°C x 3', (96°C x 30", 62°C x 30") x 35 cycles** | **179 bp** |
| **SLC17A7** | **5’-AACGTGAACCACCTGGACAT-3’** | **5’-CAGGGAGGCAATTAGGAACA-3’** | **95°C x 3', (96°C x 30", 62°C x 30") x 35 cycles** | **165 bp** |

B) Primer sequences with their corresponding annealing temperatures used to amplify the *BAHD1* and *DMRTA1* sequences for COBRA and Bio-COBRA.

| ***Gene*** | ***Forward primer*** | ***Reverse Primer*** | ***Annealing*** | ***Product size*** |
| --- | --- | --- | --- | --- |
| **DMRTA1 -391 to -131** | **5’- GGGGGTTATAGAGGTTTTTGTGTT -3’** | **5’-** **AACCAACTCACCTTATTTTTCCTCAAA -3’** | **60°C **** | **218 bp^A^** |
| **BAHD1 +296 to +499** | **5’-** **TGGTTTTTTTGAAGTTTTGTTTTGG -3’** | **5’-** **CCAATCTCCCAACTACTAACCCC C-3’** | **60°C **** | **181 bp** |

C) Primer sequences with their corresponding annealing temperatures used to amplify the *OLIG1* constructs used in the luciferase assays.

| ***Construct*** | ***Forward primer*** | ***Reverse Primer*** | ***Annealing*** | ***Product size*** |
| --- | --- | --- | --- | --- |
| **OLIG1 -21 to -267** | **5’-GCGGCCGCCGACCAGTCCTCCTTCAACAG-3’** | **5’-GATATCCTACCCCTTTAAACCCGGCTTG-3’** | **61°C *** | **246 bp** |
| **OLIG1 -21 to -564** | **5’-GCGGCCGCGAAGAGTGAATTCCAGGAACTTGG-3’** | **5’-GATATCCTACCCCTTTAAACCCGGCTTG-3’** | **61°C *** | **543 bp** |
| **OLIG1 -21 to -884** | **5’-GCGGCCGCCGACCTGAACCTGGCAGGAAATT-3’** | **5’-GATATCCTACCCCTTTAAACCCGGCTTG-3’** | **61°C *** | **863 bp** |
| **OLIG1 -21 to -1224** | **5’-GCGGCCGCCAAGTTATAGTAGCATGCACTG-3’** | **5’-GATATCCTACCCCTTTAAACCCGGCTTG-3’** | **61°C *** | **1,203 bp** |

D) Primer sequences with their corresponding annealing temperatures used to amplify the *OLIG1* regions selected for bisulfite DNA sequencing.

| ***Gene*** | ***Forward primer*** | ***Reverse Primer*** | ***Annealing*** | ***Product size*** |
| --- | --- | --- | --- | --- |
| **OLIG1 -391 to -131** | **5’-TGAGTTGTAGTTTGATTAGTG-3’** | **5’-TTTAAACCRGACTTAAAAACCTA-3’** | **56°C **** | **260 bp^A^** |
| **OLIG1 +296 to +499** | **5’-GGTTTTTAAGTYGGGTTTAAAGG-3’** | **5’-ACTACCTATAACCCACCAACTC-3’** | **50°C **** | **203 bp** |

*: The PCR condition for these reactions was 95°C x 10', [(96°Cx30", 61°Cx30", 72°Cx20") x 35] followed by a final extension at 72°C for 10'

**: The PCR condition for these reactions was 95°C x 10', [(96°Cx30", ATx30", 72°Cx20") x 35] followed by a final extension at 72°C for 10'

AT: Annealing temperature

A: Primer set also used for Bio-COBRA
