# Supplementary material for: Aberrant DNA Methylation of OLIG1, a Novel Prognostic Factor in Non-Small Cell Lung Cancer
Source: PLoS Med. 2007 Mar 27;4(3):e108. doi: 10.1371/journal.pmed.0040108 (PMC1831740; doi:10.1371/journal.pmed.0040108)
Supplement: Table S3 — (A) Adenocarcinoma samples; the age range for the sample set is indicated in brackets underneath the mean age value. (B) SCC samples; the age range for the sample set is indicated in brackets underneath the mean age value. (38 KB DOC) [file pmed.0040108.st003.doc]

Table S3

Clinical characteristics of the subset of tumor samples present in tissue array 1 (TMA1) which met all the quality control criteria to be included in the analysis for OLIG1 protein expression.

A) Clinical features of the adenocarcinoma samples included in the array. The age range

for the sample set is indicated in brackets underneath the mean age value.

| ***Total cases*** | ***Gender distribution*** | | ***T stage*** | | ***N stage*** | | ***M stage*** | | ***Mean age*** | |
| --- | --- | --- | --- | --- | --- | --- | --- | --- | --- | --- |
| **59** | **Males** | **58%** | **T 1** | **36%** | **N 0** | **78%** | **M 0** | **93%** | **61** | |
|  | **Females** | **42%** | **T 2** | **57%** | **N 1** | **22%** | **M 1** | **7%** | **(32-83)** |  |
|  |  |  | **T 3** | **7%** | **N 2** | **0%** |  |  |  |  |

B) Clinical features of the squamous cell carcinoma samples included in the tissue array.

The age range for the sample set is indicated in brackets underneath the mean age

value.

| ***Total cases*** | ***Gender distribution*** | | ***T stage*** | | ***N stage*** | | ***M stage*** | | ***Mean age*** | |
| --- | --- | --- | --- | --- | --- | --- | --- | --- | --- | --- |
| **74** | **Males** | **68%** | **T 1** | **34%** | **N 0** | **81%** | **M 0** | **100%** | **62** | |
|  | **Females** | **32%** | **T 2** | **53%** | **N 1** | **12%** | **M 1** | **0%** | **(34-82)** |  |
|  |  |  | **T 3** | **13%** | **N 2** | **7%** |  |  |  |  |
