# Supplementary material for: Aberrant DNA Methylation of OLIG1, a Novel Prognostic Factor in Non-Small Cell Lung Cancer
Source: PLoS Med. 2007 Mar 27;4(3):e108. doi: 10.1371/journal.pmed.0040108 (PMC1831740; doi:10.1371/journal.pmed.0040108)
Supplement: Table S5 — (A) Primer sequences and PCR conditions used to evaluate mRNA expression in the genes listed on the left. The OLIG1 and CAMKK2 primers were also used to assess for OLIG1 deletions in primary tumors. (B) Primer sequences with their corresponding annealing temperatures used to amplify the BAHD1 and DMRTA1 for Bio-COBRA. (C) Primer sequences with their corresponding annealing temperatures used to amplify the OLIG1 constructs used in the luciferase assays. (D) Primer sequences with their corresponding annealing temperatures used to amplify the OLIG1 regions selected for bisulfite DNA sequencing. (146 KB DOC) [file pmed.0040108.st005.doc]

Table S5

Average OLIG1 index for each of the samples used for immunohistochemistry in tissue array 1 (TMA). Samples are classified into 3 categories: positive (index >6), negative (index<4) and low level (index 4-5)

| ***Sample*** | ***TMA index average***  ***(4 cores)*** | ***OLIG1***  ***status*** |  | ***Sample*** | ***TMA index average***  ***(4 cores)*** | ***OLIG1***  ***status*** |
| --- | --- | --- | --- | --- | --- | --- |
| **1** | **2** | **Negative** |  | **68** | **2** | **Negative** |
| **2** | **4** | **Low level** |  | **69** | **1** | **Negative** |
| **3** | **3** | **Negative** |  | **70** | **6** | **Positive** |
| **4** | **3** | **Negative** |  | **71** | **2** | **Negative** |
| **5** | **5** | **Low level** |  | **72** | **5** | **Low level** |
| **6** | **4** | **Low level** |  | **73** | **3** | **Negative** |
| **7** | **6** | **Positive** |  | **74** | **6** | **Positive** |
| **8** | **1** | **Negative** |  | **75** | **6** | **Positive** |
| **9** | **2** | **Negative** |  | **76** | **2** | **Negative** |
| **10** | **1** | **Negative** |  | **77** | **5** | **Low level** |
| **11** | **0** | **Negative** |  | **78** | **5** | **Low level** |
| **12** | **0** | **Negative** |  | **79** | **7** | **Positive** |
| **13** | **2** | **Negative** |  | **80** | **9** | **Positive** |
| **14** | **1** | **Negative** |  | **81** | **4** | **Low level** |
| **15** | **2** | **Negative** |  | **82** | **8** | **Positive** |
| **16** | **6** | **Positive** |  | **83** | **6** | **Positive** |
| **17** | **3** | **Negative** |  | **84** | **4** | **Low level** |
| **18** | **2** | **Negative** |  | **85** | **4** | **Positive** |
| **19** | **5** | **Low level** |  | **86** | **8** | **Positive** |
| **20** | **5** | **Low level** |  | **87** | **4** | **Low level** |
| **21** | **5** | **Low level** |  | **88** | **6** | **Positive** |
| **22** | **5** | **Low level** |  | **89** | **3** | **Negative** |
| **23** | **3** | **Negative** |  | **90** | **3** | **Negative** |
| **24** | **3** | **Negative** |  | **91** | **4** | **Low level** |
| **25** | **2** | **Negative** |  | **92** | **4** | **Low level** |
| **26** | **8** | **Positive** |  | **93** | **6** | **Positive** |
| **27** | **1** | **Negative** |  | **94** | **9** | **Positive** |
| **28** | **4** | **Low level** |  | **95** | **2** | **Negative** |
| **29** | **6** | **Positive** |  | **96** | **5** | **Low level** |
| **30** | **3** | **Negative** |  | **97** | **7** | **Positive** |
| **31** | **2** | **Negative** |  | **98** | **8** | **Positive** |
| **32** | **6** | **Positive** |  | **99** | **8** | **Positive** |
| **33** | **8** | **Positive** |  | **100** | **5** | **Low level** |
| **34** | **2** | **Negative** |  | **101** | **9** | **Positive** |
| **35** | **2** | **Negative** |  | **102** | **6** | **Positive** |
| **36** | **7** | **Positive** |  | **103** | **4** | **Low level** |
| **37** | **6** | **Positive** |  | **104** | **3** | **Negative** |
| **38** | **7** | **Positive** |  | **105** | **8** | **Positive** |
| **39** | **9** | **Positive** |  | **106** | **6** | **Positive** |
| **40** | **5** | **Low level** |  | **107** | **2** | **Negative** |
| **41** | **7** | **Positive** |  | **108** | **4** | **Low level** |
| **42** | **3** | **Negative** |  | **109** | **4** | **Low level** |
| **43** | **6** | **Positive** |  | **110** | **4** | **Low level** |
| **44** | **4** | **Low level** |  | **111** | **7** | **Positive** |
| **45** | **4** | **Low level** |  | **112** | **6** | **Positive** |
| **46** | **3** | **Negative** |  | **113** | **8** | **Positive** |
| **47** | **3** | **Negative** |  | **114** | **6** | **Positive** |
| **48** | **1** | **Negative** |  | **115** | **8** | **Positive** |
| **49** | **0** | **Negative** |  | **116** | **6** | **Positive** |
| **50** | **3** | **Negative** |  | **117** | **1** | **Negative** |
| **51** | **6** | **Positive** |  | **118** | **3** | **Negative** |
| **52** | **5** | **Low level** |  | **119** | **4** | **Low level** |
| **53** | **5** | **Low level** |  | **120** | **6** | **Positive** |
| **54** | **4** | **Low level** |  | **121** | **3** | **Negative** |
| **55** | **2** | **Negative** |  | **122** | **3** | **Negative** |
| **56** | **5** | **Low level** |  | **123** | **4** | **Low level** |
| **57** | **1** | **Negative** |  | **124** | **5** | **Low level** |
| **58** | **3** | **Negative** |  | **125** | **2** | **Negative** |
| **59** | **3** | **Negative** |  | **126** | **8** | **Positive** |
| **60** | **4** | **Low level** |  | **127** | **5** | **Low level** |
| **61** | **2** | **Negative** |  | **128** | **0** | **Negative** |
| **62** | **5** | **Low level** |  | **129** | **7** | **Positive** |
| **63** | **6** | **Positive** |  | **130** | **4** | **Low level** |
| **64** | **6** | **Positive** |  | **131** | **8** | **Positive** |
| **65** | **7** | **Positive** |  | **132** | **8** | **Positive** |
| **66** | **3** | **Negative** |  | **133** | **2** | **Negative** |
| **67** | **3** | **Negative** |  |  |  |  |
